# Solid lipid nanoparticle as an effective drug delivery system of a novel curcumin derivative: formulation, release *in vitro* and pharmacokinetics *in vivo*

**DOI:** 10.1080/13880209.2022.2136205

**Published:** 2022-11-23

**Authors:** Wenmei Zhao, Mingtang Zeng, Ke Li, Chao Pi, Zerong Liu, Chenglin Zhan, Jiyuan Yuan, Zhilian Su, Yuxun Wei, Jie Wen, Fengjuan Pi, Xinjie Song, Robert J. Lee, Yumeng Wei, Ling Zhao

**Affiliations:** a Key Laboratory of Medical Electrophysiology, Ministry of Education, School of Pharmacy of Southwest Medical University, Luzhou, PR China; b Luzhou Key Laboratory of Traditional Chinese Medicine for Chronic Diseases Jointly Built by Sichuan and Chongqing, The Affiliated Traditional Chinese Medicine Hospital of Southwest Medical University, Luzhou, PR China; c Central Nervous System Drug Key Laboratory of Sichuan Province, Southwest Medical University, Luzhou, PR China; d Central Nervous System Drug Key Laboratory of Sichuan Province, Sichuan Credit Pharmaceutical Co., Ltd., Luzhou City, PR China; e Key Laboratory of Biorheological Science and Technology, Ministry of Education, College of Bioengineering, Chongqing University, Chongqing, PR China; f Department of Pharmacy, The Affiliated Hospital of Southwest Medical University, Luzhou, PR China; g Clinical Trial Center, The Affiliated Traditional Chinese Medicine Hospital of Southwest Medical University, Luzhou, PR China; h Department of Pharmacy, The Traditional Chinese Medicine Hospital of Luzhou, Luzhou, PR China; i School of Biological and Chemical Engineering, Zhejiang University of Science and Technology, Hangzhou, China; j Department of Food Science and Technology, Yeungnam University, Gyeongsan, Republic of Korea; k Division of Pharmaceutics and Pharmacology, College of Pharmacy, The Ohio State University, Columbus, OH, USA

**Keywords:** Small molecule derivative of curcumin, Poloxamer 188, nanotechnology

## Abstract

**Context:**

Curcumin (Cur) has a short duration of action which limits its therapeutic efficacy. Carbonic acid 17-(1,5-dimethyl-hexyl)-10,13-dimethyl-2,3,4,7,8,9,10,11,12,13,14,15,16,17-tetradecahydro-1H-cyclopenta[a]phenanthren-3-yl ester 4-[7-(4-hydroxy-3-methoxy-phenyl)-3,5-dioxo-hepta-1,6-dienyl]-2-methoxy-phenyl ester (CUD), as a small molecule derivative of Cur with superior stability, has been developed in our laboratory.

**Objective:**

CUD-loaded solid lipid nanoparticles (CUD-SLN) were prepared to prolong the duration of the drug action of Cur.

**Materials and methods:**

CUD-SLN were prepared with Poloxamer 188 (F68) and hydrogenated soybean phospholipids (HSPC) as carriers, and the prescription was optimized. The *in vitro* release of CUD and CUD-SLN was investigated. CUD-SLN (5 mg/kg) was injected into Sprague Dawley (SD) rats to investigate its pharmacokinetic behaviour.

**Results:**

CUD-SLN features high entrapment efficiency (96.8 ± 0.4%), uniform particle size (113.0 ± 0.8 nm), polydispersity index (PDI) (0.177 ± 0.007) and an appropriate drug loading capacity (6.2 ± 0.1%). Optimized CUD-SLN exhibited sustained release of CUD for about 48 h. Moreover, the results of the pharmacokinetic studies showed that, compared to Cur, CUD-SLN had a considerably prolonged half-life of 14.7 h, slowed its metabolism *in vivo* by 35.6-fold, and had an improved area under the curve (AUC_0–_*_t_*) of 37.0-fold.

**Conclusions:**

CUD-SLN is a promising preparation for the development of a small molecule derivative of Cur.

## Introduction

Curcumin (Cur), a natural polyphenolic compound, can be found in the tuber or rhizome of *Curcuma longa* L. (Zingiberaceae), has been widely used in traditional Chinese medicine. It has been reported that Cur has several valuable pharmacological properties, including antibacterial, anti-inflammatory, antitumor and neuroprotection activities (Feng T et al. 2017; Momtazi-Borojeni et al. 2018; Bagheri et al. 2020; Lyu et al. 2020; Cai et al. 2021), thereby showing great potential for the development of therapeutics. However, the short duration of drug action has seriously hindered the efficacy of Cur. *In vivo*, Cur will rapidly incur reduction and bind with glucuronic acid; the metabolites are mainly dihydrocurcumin, tetrahydrocurcumin, hexahydrocurcumin, octahydrocurcumin and tetrahydrocurcumin-glucuronide (Shi et al. 2019). The major reason contributing to the rapid metabolism is the poor stability of all Cur metabolites (Ma et al. 2019; Slika and Patra 2020). Therefore, Cur usually requires frequent dosing.

To prolong the duration of drug action, the current study has mainly focussed on the modification of the structure of Cur and the development of new Cur-based preparations. Cur contains two phenolic hydroxyl groups (PhOH), which are key sites for metabolism and potential sites for binding with biological macromolecules. Therefore, the PhOH groups of Cur could be modified to improve the *in vivo* stability and ameliorate the pharmacokinetic behaviour of Cur (Pawar et al. 2016; Chen S et al. 2018; Zhao et al. 2018; Chainoglou and Hadjipavlou-Litina 2019). For instance, a succinate prodrug of Cur was synthesized by ester-forming modification of the 4′-hydroxyl group of Cur, which remarkably improved the chemical stability and increased the half-life (*t*_1/2_) to 7.66 h (Wichitnithad et al. 2011). Cur was reacted with demethylated piperonyl chloride in pyridine to obtain Cur-piperine derivatives of diesters, and piperine which, when generated during metabolism increased the area under the curve (AUC_0–_*_t_*) of Cur in rats by nearly 20-fold (Mishra et al. 2005; Dubey et al. 2008). To prolong the duration of the drug action of Cur, several possible drug delivery systems have been investigated, including polymer-drug micelles, microspheres and supersaturated self-nano-emulsion (Tian et al. 2019; Ban et al. 2020). Compared with free Cur, redox sensitive hyaluronic acid (HA)-ss-Cur micelles had about a 4.70-fold higher AUC_0–_*_t_* after intravenous injection (Tian et al. 2019). Moreover, Cur Microspheres containing ascorbic acid (AA) had about a seven-fold increase in plasma concentration (Karade and Jadhav 2018). Cur supersaturated self-nano-emulsion prepared with 2% HPMC55-60 as a precipitation inhibitor increased the AUC_0–12h_ by 3.50-fold (Chen XL et al. 2021).

Solid lipid nanoparticle (SLN) is a class of drug delivery system which was developed in the early 1990s (Yingchoncharoen et al. 2016). SLN is mainly composed of lipids which are solid at room temperature and body temperature, and surfactants can help SLN become more stable. Compared with polymer nanoparticles, SLN is mainly made of physiologically tolerated lipid components, thereby reducing the possibility of acute or chronic poisoning (Iqra et al. 2021; Mohammed et al. 2022). Compared with liposomes, SLN has a better stability and fewer drug leakage problems (Alam et al. 2020).

**Figure 1.**
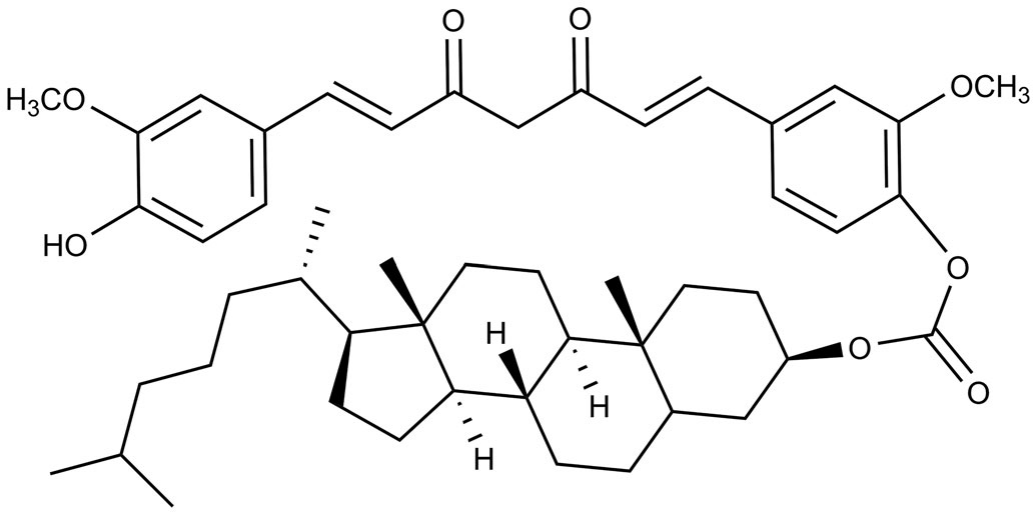
The chemical structure of CUD.

To further prolong the duration of drug action of Cur, carbonic acid 17-(1,5-dimethyl-hexyl)-10,13-dimethyl-2,3,4,7,8,9,10,11,12,13,14,15,16,17-tetradecahydro-1*H*-cyclopenta[*a*]phenanthren-3-yl ester 4-[7-(4-hydroxy-3-methoxy-phenyl)-3,5-dioxo-hepta-1,6-dienyl]-2-methoxy-phenyl ester, named CUD (Figure 1) (98% purity), was synthesized in our laboratory (Wei et al. 2022). CUD, a small molecule derivative of Cur with superior stability, was chosen to develop solid lipid nanoparticles (CUD-SLN) composed of hydrogenated soybean phospholipids (HSPC), Poloxamer 188 (F68) and sucrose. CUD-SLN was prepared using a thin-film ultrasonic dispersion technology, and the physicochemical properties, drug release behaviour *in vitro*, and pharmacokinetics *in vivo* of CUD-SLN were evaluated.

## Materials and methods

### Materials

Cur (purity > 98%), cholesteryl chloroformate (RoCL), triethylamine (TEA), isopropanol, dichloromethane (anhydrous), HSPC, Poloxamer 188 (F68) and sucrose were purchased from Luzhou Renkang Biotechnology Co., Ltd. (Luzhou, China). CUD was obtained from our laboratory.

### Animals

Sprague Dawley (SD) male rats aged 8 weeks weighing 250–300 g were provided by the Laboratory Animal Center of Southwest Medical University and were placed in a pathogen-free environment, and had free access to food and water. Studies involving animals were approved (approval number: SCXK (Chuan) 2013-17) by the Ethics Committee of Southwest Medical University.

### Ultraviolet–visible (UV–vis) absorption spectrum

UV wavelength scans of Cur and CUD from 700 to 200 nm were recorded at 25 °C using a UV spectrophotometer (A390, Aoyi Instruments Shanghai Co., Ltd., Shanghai, China).

### Preparation of CUD-SLN

CUD-SLN was prepared by using film ultrasonic dispersion technology (Li W et al. 2014). In brief, CUD, HSPC and F68 were dissolved in 2 mL organic solvent by the method of ultrasound. Then, the solution was transferred to a beaker (50 mL) and was stirred to become a film at 30 °C. Deionized water (30 mL) containing 1.25% sucrose was added to the beaker, and sonicated until dissolved. Then, the solution was homogenized by a high-pressure homogenizer (AH-100D; ATS Automation Tooling Systems Inc., Cambridge, Canada). The final solution was lyophilized by freeze-dryer (LGJ-18C; Fourth-Ring Science Instrument Plant Beijing Co., Ltd., Beijing, China) at −50 °C 0.9 Pa for 24 h to obtain CUD-SLN.

### Optimization of CUD-SLN

The effect of temperature (films were prepared), the input ratio of HSPC:F68 (w/w), and CUD:(HSPC + F68) (w/w) on particle size, polydispersity index (PDI) and encapsulation efficiency (EE%) of CUD-SLN were investigated by the single factor method. The specific formulations are listed in Table 1. In addition, the protective abilities of sucrose with different concentrations as lyoprotectants of CUD-SLN were evaluated (Table 2).

**Table 1.** Single-factor investigation results (*n* = 3).

No.	Temperature (°C)	HSPC:F68	CUD:(HSPC + F68)	Particle size (nm)	PDI	EE (%)
*Optimization of temperature*						
F1	30	5:7	1:4	107.8 ± 2.3	0.186 ± 0.027	97.3 ± 2.1
F2	40	5:7	1:4	102.0 ± 1.7	0.176 ± 0.015	95.6 ± 3.2
F3	50	5:7	1:4	101.9 ± 2.5	0.183 ± 0.012	93.0 ± 2.7
F4	60	5:7	1:4	107.7 ± 1.4	0.182 ± 0.022	92.8 ± 2.1
*Optimization of HSPC:F68*						
F5	30	3:7	1:4	100.7 ± 1.3	0.186 ± 0.021	98.3 ± 2.4
F6	30	7:7	1:4	95.8 ± 2.7	0.197 ± 0.015	93.0 ± 1.7
F7	30	11:7	1:4	90.4 ± 2.3	0.192 ± 0.011	90.4 ± 2.5
*Optimization of CUD:(HSPC + F68)*						
F8	30	3:7	1:1	124.7 ± 5.2	0.156 ± 0.042	58.6 ± 4.9
F9	30	3:7	1:3	124.4 ± 1.9	0.143 ± 0.025	97.3 ± 2.1
F10	30	3:7	1:5	124.8 ± 3.8	0.137 ± 0.031	96.5 ± 1.3
F11	30	3:7	1:7	127.8 ± 2.3	0.122 ± 0.024	97.4 ± 1.1

EE: entrapment efficiency; PDI: polydispersity index.

**Table 2.** The effects of lyoprotectant ratio on particle size and PDI after freeze drying (*n* = 3).

Sucrose content (w/v)	Particle size (nm)		PDI	
	Before	After	Before	After
0.5%	110.1 ± 2.9	188.3 ± 3.7	0.136 ± 0.024	0.379 ± 0.070
1.25%	109.4 ± 3.1	115.6 ± 2.3	0.146 ± 0.033	0.161 ± 0.014
2.5%	110.3 ± 2.5	115.9 ± 4.2	0.149 ± 0.014	0.142 ± 0.016
5.0%	110.2 ± 1.7	166.8 ± 2.8	0.158 ± 0.028	0.293 ± 0.022

PDI: polydispersity index.

Based on the results of the single-factor study, a comprehensively designed experiment was performed to optimize the formulation of CUD-SLN. Two factors of CUD-SLN including the input ratio of CUD:(HSPC + F68) (A) and HSPC:F68 (B) (each factor had three levels) were arranged according to an L9 (3^2^) comprehensively designed experiment (Table 3). The EE% and drug loading (DL%), which are important indicators of SLN quality, were selected as the evaluation index. Intuitive and variance analyses were used to identify the best formulation.

**Table 3.** The formulations and the results of EE% and DL% (*n* = 3).

No.		CUD:(HSPC + F68)	HSPC:F68	EE (%)	DL (%)
1		1:2	4:7	90.4 ± 1.6	5.9 ± 0.1
2		1:2	6:7	86.4 ± 2.3	5.3 ± 0.2
3		1:2	8:7	86.9 ± 1.4	5.0 ± 0.1
4		1:3	4:7	96.8 ± 0.4	6.3 ± 0.1
5		1:3	6:7	89.7 ± 3.9	5.5 ± 0.2
6		1:3	8:7	92.8 ± 2.6	5.3 ± 0.2
7		1:4	4:7	95.6 ± 1.9	6.2 ± 0.1
8		1:4	6:7	84.2 ± 6.2	5.2 ± 0.4
9		1:4	8:7	87.7 ± 1.6	5.0 ± 0.1
EE (%)	K1	263.7	282.8		
	K2	279.3	260.3		
	K3	267.5	267.4		
	R	15.6	22.5		
DL (%)	K1	16.2	18.4		
	K2	17.1	16.0		
	K3	16.4	15.3		
	R	0.9	3.1		

DL: drug loading; EE: entrapment efficiency.

### Confirmatory test

To verify whether the optimized formulation process was stable and feasible, three batches of CUD-SLN samples were prepared, and the EE%, DL%, particle size, PDI and zeta potential of the three batches tested.

### Characterization of CUD-SLN

#### Particle size, PDI and zeta potential

CUD-SLN (nanoparticles with a concentration of 10 mg/mL) were characterized for particle size, PDI and zeta potential by a Malvern’s Zetasizer Nano ZS instrument (ZS; Malvern Instruments, Malvern, UK). CUD-SLN was measured three times in a sample cell (1 mL), and the light scattering angle was set to 90°.

#### Encapsulation efficiency and drug loading

CUD is a hydrophobic molecule. Hence, CUD-SLN with smaller particle sizes cannot form a precipitate under low-speed and medium-speed centrifugation, while the drug in the free state will be precipitated in crystal form. Therefore, to evaluate the EE%, low-speed centrifugation (8000 rpm, 8 min) was selected to separate free CUD. The same volume of SLN suspension before and after low-speed centrifugation was measured, demulsified and diluted with solvent (methanol–ethyl acetate 10:90, v/v). Lyophilized powder of SLN was accurately weighed, dissolved in methanol, and demulsified by ultrasound (200 W, 40 kHz, 4 min) to ensure the complete release of all drugs. After the sample was filtered and diluted with solvent (methanol–ethyl acetate 10:90, v/v), the concentration of CUD was determined by UV–vis spectrophotometer at 409 nm, using a standard curve (*A* = 0.0495*C* – 0.0061, *R*^2^=0.9998). The formulas used for the calculation of the EE% and DL% were as follows:

(1) $$\mathrm{Encapsulation}\;(\text{EE\%})=\frac{W_{e}}{W_{t}}\times 100\%\;$$

(2) $$\text{Drug loading }(\text{DL\%})=\frac{W_{t}}{W_{0}}\times 100\%\;$$

where *W*_e_ is the amount of drug loaded in CUD-SLN, *W*_t_ is the total amount of drug in CUD-SLN and *W*_0_ is the total weight of SLN.

#### Differential scanning calorimetry

The purpose of differential scanning calorimetry (DSC) was to study the physical integrity of the drug in the SLN. The DSC thermograms of the CUD, blank-SLN and CUD-SLN were obtained by microcalorimeter (NETZSCH TG-DSC STA-449 F3, Bavaria, Germany). Samples were heated in the temperature range of 30–400 °C on an aluminium pan at a rate of 10 °C/min, under nitrogen atmosphere.

#### X-ray diffraction

X-ray diffraction (XRD) analysis was performed to monitor changes in crystallization characteristics of the drug when CUD was loaded into SLN. The XRD patterns of CUD, blank-SLN and CUD-SLN were measured using an X*'* D/MAX-2500/PC diffract metre (Rigaku Corporation, Tokyo, Japan).

#### Transmission electron microscopy

Solid freeze-dried nanoparticles were dissolved in ultrapure water, dripped on a slide, and a negative stain was performed with 2% phosphotungstic acid. Sample was placed on a copper grid until dried. The morphology of CUD-SLN was observed by transmission electron microscope (TEM) (microscopyH-7500; Hitachi Ltd., Tokyo, Japan).

### *In vitro* drug release profile

The release of CUD-SLN and free CUD *in vitro* was studied using a dialysis membrane diffusion technique (MWCO 8000–14,000) (He et al. 2020). Due to the low solubility of CUD in the buffer, Tween 80 was added to satisfy the leaking conditions. A total of 500 mL of PBS buffer with 10% w/v Tween 80 (pH 7.35) was used as a release medium. Next, 2 mL of freshly prepared CUD-SLN (1 mg/mL) was transferred to the dialysis bags, and 2 mg free CUD (suspended in 2 mL 0.5% w/v CMC-Na) was transferred to dialysis bags as a control. The test bags were immersed in a release medium at 37 ± 0.5 °C and stirred at 100 rpm using a dissolution apparatus (ZRS-8G, Intelligent dissolution tester, ZRS-8G, Tianjin, China). An aliquot of the sample (1 mL) was removed from the release medium at 15 and 30 min and 1, 2, 4, 6, 8, 12, 24 and 48 h. After each sampling, the blank dissolution medium was replenished with the same temperature and volume. Removed samples were filtered through a 0.22 μm filter, the CUD content was analysed by a UV–vis spectrophotometer, and the analytical method described before. The *in vitro* release data of the drug were fitted with the zero-order release equation, the first-order release equation and the Higuchi equation (Ma et al. 2019).

### Pharmacokinetic studies

Rats that were used for the pharmacokinetic study were fasted for more than 12 h before use and had free access to water. Rats were randomly divided into two groups (*n* = 5), namely, the Cur group and CUD-SLN group, Rats in both groups were injected intravenously with 5 mg/kg of corresponding drugs. Approximately, 200 µL of blood was collected using a heparin-coated syringe at 2, 5, 15 and 30 min and at 1, 2, 4, 6, 8, 12, 24, 30, 36, 48, 54, 60, 72 and 84 h after injection. Blood was centrifuged for 10 min at 3000 rpm, and plasma was obtained. Furthermore, 50 µL of acetate buffer (pH 3.5) was added to 100 μL of plasma. After vortexing for 1 min, 0.5 mL of extract solvent (methanol–ethyl acetate 10:90, v/v) was added to the mixture, the mixture was vortexed again for 3 min, followed by centrifugation for 3 min at 8000 rpm. Then, 0.5 mL of supernatant was removed and evaporated with nitrogen flow. Next, 200 μL of reconstituted solvent (acetonitrile contains 0.1% phosphoric acid) was added to the dry residue, vortexed for 4 min, sonicated for 4 min, and centrifuged for 10 min at 10,000 rpm, A total of 50 μL of the supernatant was removed for the high-performance liquid chromatography (HPLC) (1260 Infinity II, Agilent Technologies, Santa Clara, CA) and calculated with the standard curve (Cur: *A* = 157.21 C + 0.148, *R*^2^=0.9991, range: (0.02, 12) μg/mL; CUD: *A* = 18.159 C + 0.4884, *R*^2^=0.9998, range: (0.02, 5) μg/mL; *A* = 46.552 C – 179.81, *R*^2^=0.9997, range: (5, 160) μg/mL). Separation was performed on a reverse-phase C18 column (5 μm, 4.6 × 150 mm) at 25 °C and a mobile phase flow rate of 1.0 mL/min. The detection wavelength was 409 nm (Guo et al. 2020).

### Statistical analysis

Evaluation of the comprehensive design experiment was performed by SPSS 17.0 software (SPSS Inc., Chicago, IL). Fitting of the release curve was performed by Origin 8.0 software. Pharmacokinetic data were processed by DAS 2.1.1 software to obtain relevant pharmacokinetic parameters. Student’s *t*-test for two groups and one-way ANOVA for multiple groups were employed for the data analysed. *p* < 0.05 was considered statistically significant.

## Results and discussion

### The UV–vis absorption spectrum of CUD

The metabolic processes of Cur *in vivo* included oxidation, reduction, glycosylation and sulphation. PhOH is the key site of glycosylation and sulphation, and significantly influences the metabolism processes and the stability of Cur *in vivo*. Therefore, the modification of PhOH can improve the stability of Cur *in vivo*. *In vivo*, drugs need to penetrate cell membranes to work. Cholesterol is regarded as an important component of the cell membrane. Thus, taking the active 4-OH group of Cur as the site, through covalent bonding with cholesterol, new derivatives are formed, which can improve the permeability of Cur to the cell membrane, enhance the stability of Cur *in vivo*, and improve the efficacy (Dubey et al. 2008).

**Figure 2.**
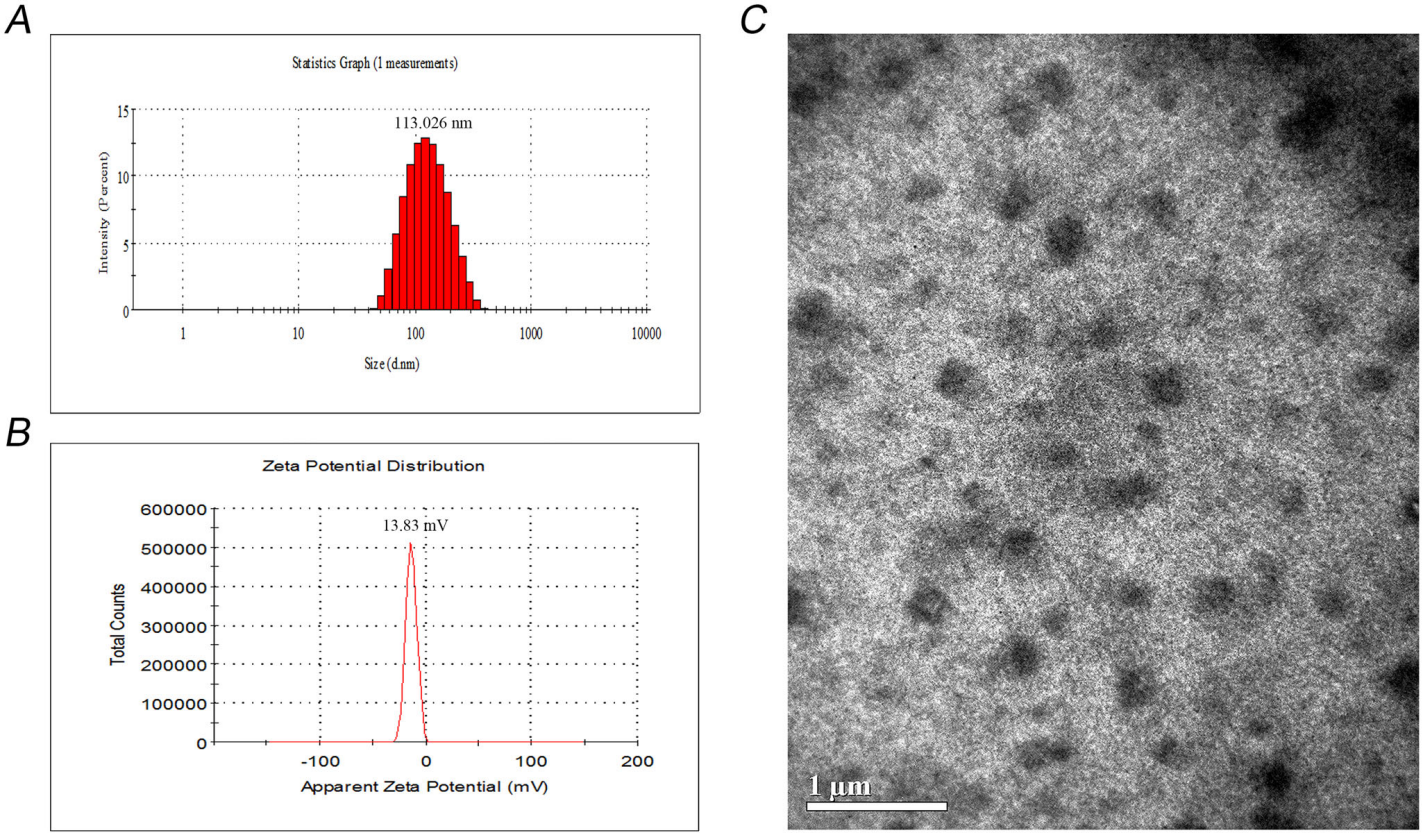
UV–vis absorption spectrum of Cur and CUD (A). DSC thermograms of CUD, blank SLN and CUD-SLN (B). XRD behaviour of CUD, blank SLN and CUD-SLN (C).

As shown in Figure 2(A), CUD has a maximum absorption at a wavelength of 409 nm, while Cur has a maximum absorption at 425 nm. This phenomenon originates from the electron-withdrawing groups replacing the phenolic hydroxyl group in Cur, result in a significant blue-shift in the UV–vis absorption spectrum.

### Preparation and optimization of CUD-SLN

In this study, various factors that may affect the quality of CUD-SLN were investigated. CUD-SLN was composed of HSPC (solid lipid material), F68 (non-ionic surfactant) and sucrose (lyoprotectant). As shown in Table 1, when the temperature was 30 °C (F1), the EE% was the highest, and no significant differences were observed between the particle size and PDI. The EE% was significantly influenced by changes in the volatilization temperature, and with an increase in temperature, the EE% showed a downward trend. This may be due to the accelerated evaporation of the solvent with the increase of temperature, resulting in precipitation of the drug, leading to a decrease in EE%. Therefore, 30 °C was chosen as the volatilization temperature for subsequent experiments.

Formulations F5–F7 showed that with the increase in the ratio HSPC:F68, the particle size decreased, but this change was not significant. The EE% increased with a decrease in the ratio HSPC:F68. When the HSPC:F68 ratio was 3:7 (F5), the EE% was the highest at 98.3 ± 2.4%. The amount of surfactant F68 may play a crucial role in stabilizing CUD-SLN; however, excessive F68 may not be induced to film formation of the lipid material, thereby reducing the EE% of the drug. Therefore, the HSPC:F68 ratio was temporarily set to 3:7 for subsequent experiments. The EE% of CUD-SLN was significantly influenced by the content of the excipients in the formula, and the particle size and PDI hardly changed with a change in the drug excipients ratio. When the drug excipients ratio was reduced from 1:7 to 1:3, no significant changes were observed in EE%. However, when the adjuvant ratio was decreased to 1:1, the EE% decreased sharply to 58.6 ± 4.9%. To obtain a higher DL% with lower excipients, a drug excipients ratio of 1:3 was used for subsequent experiments.

Based on a preliminary experiment, it was found that the amount and type of lyophilized protectants had the greatest effect on particle size and PDI of CUD-SLN. In this study, sucrose was chosen as a lyophilized protectants and the results are presented in Table 2. When the content of sucrose was 1.25% and 2.5% in the formulations of the lyophilized product, small changes of particle size and PDI of CUD-SLN before and after freeze-drying were observed. To increase the DL%, 1.25% of sucrose was selected for further development.

Based on the single-factor test, two main factors affecting the quality of CUD-SLN were identified, namely HSPC:F68 (A) and CUD:(HSPC + F68) (B), and each factor has three levels. L (3^2^) comprehensive design experiments were performed to determine a stable and feasible technological formula. The results of DL% and EE% are presented in Table 3. Furthermore, range analysis was employed to compare the effects of two factors on the EE% and DL%. The data showed that factor A had a great effect on EE% and DL%, and the best prescription was A2B1. The analysis of variance showed that factor A had a significant effect on EE% and DL% with *p* values of 0.021 and 0.001, respectively. Moreover, factor B had no significant effect on EE% and DL% (*p* values were 0.063 and 0.053, respectively), which was consistent with the results of the intuitive analysis. Taken together, the optimal proportion of CUD, HSPC and F68 in the formulation was 11:12:21.

### Validation of optimal prescription

**Figure 3.**
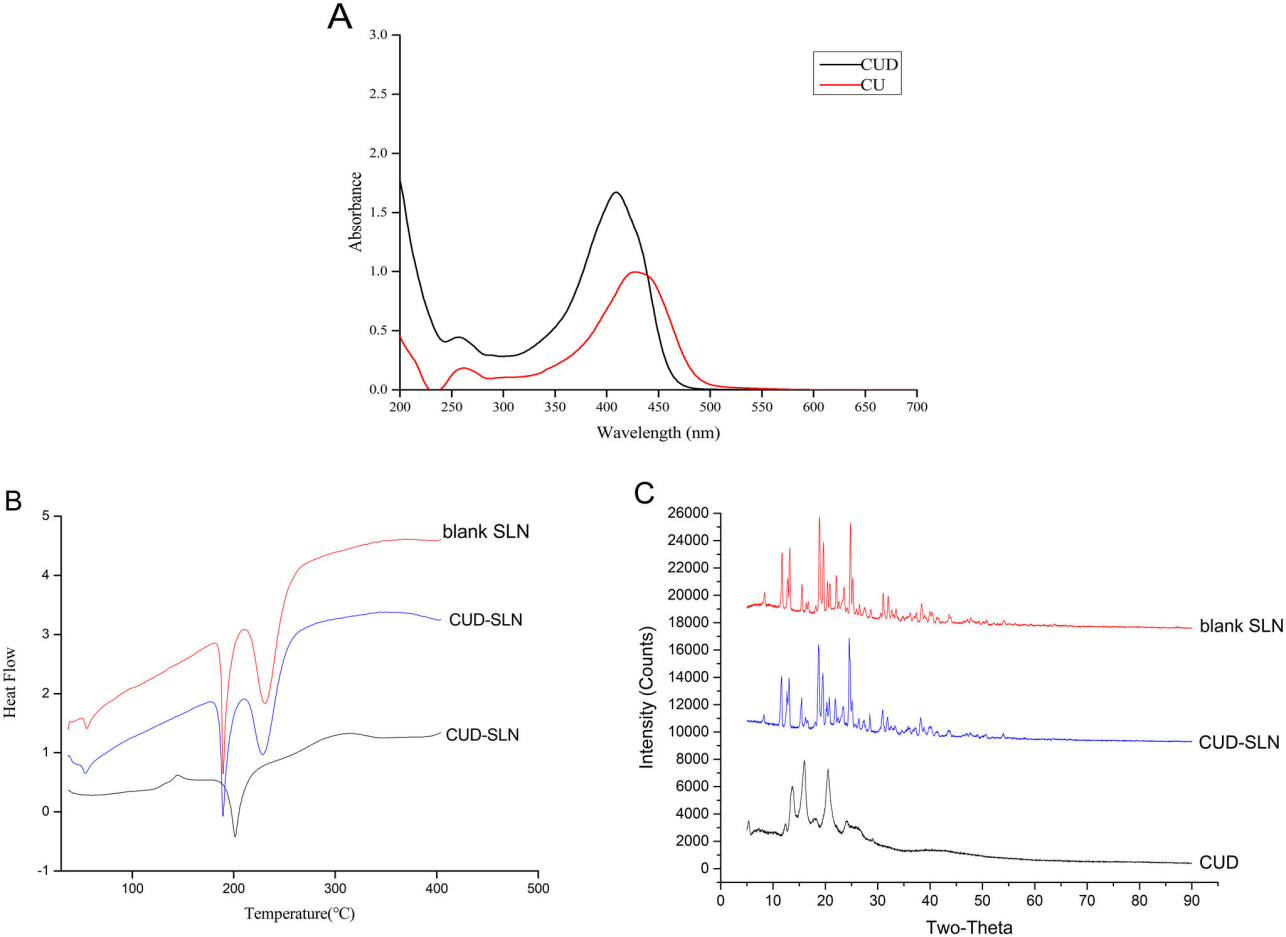
Particle size distribution of CUD-SLN (A), zeta potential distribution of CUD-SLN (B) and transmission electron microscopy of CUD-SLN (C).

To investigate whether the optimal prescription process is stable and feasible, three batches of CUD-SLN were prepared by the optimized formulation, and the EE%, DL%, PDI, particle size and zeta potential were measured and are presented in Table 4. The results indicated that the mean particle size of the three batches was 113.0 ± 0.8 nm and the PDI was 0.177 ± 0.007 (Figure 3(A)), the particle size distribution of CUD-SLN was relatively uniform, thereby indicating that CUD-SLN has good homogeneity without aggregation or fusion. The average zeta potential was −13.82 ± 0.27 mV (Figure 3(B)), which makes the formula more stable (Maritim et al. 2021). The EE% of the three batches was higher than 95%, and the DL% was higher than 6%. No significant differences were observed in the indicators of the three batches (*p* > 0.05), thus indicating that the optimized formulation method was stable and feasible.

**Table 4.** DL%, EE%, particle size, zeta potential and PDI of three batches of CUD-SLN (*n* = 3).

Parameters	Batches			
	1	2	3	Mean
DL (%)	6.3 ± 0.2	6.2 ± 0.1	6.2 ± 0.2	6.2 ± 0.1
EE (%)	96.4 ± 0.6	97.2 ± 0.9	96.7 ± 0.2	96.8 ± 0.4
Particle size (nm)	112.4 ± 2.5	113.9 ± 1.3	112.7 ± 2.7	113.0 ± 0.8
PDI	0.182 ± 0.016	0.179 ± 0.007	0.169 ± 0.011	0.177 ± 0.007
Zeta potential	–13.60 ± 0.33	–13.65 ± 0.42	–14.20 ± 0.68	–13.81 ± 0.27

DL: drug loading; EE: entrapment efficiency; PDI: polydispersity index.

### Characterization of CUD-SLN

The TEM image of CUD-SLN is shown in Figure 3(C). The SLN had a regular spherical shape, which may be because the SLN was composed of phospholipids that contain saturated fatty acids (such as HSPC and DSPC) (Tan et al. 2014). DSC thermograms of CUD, blank SLN and CUD-SLN are presented in Figure 2(B). The CUD thermogram displayed a sharp endothermic peak at 201.52 °C that corresponded to the melting point of the crystalline form. This peak disappeared in the DSC thermogram of CUD-SLN, which indicated that CUD was present as an amorphous form in CUD-SLN. XRD was used to study the changes in crystallinity characteristics of the drug when the drug was loaded into SLN. The XRD patterns of CUD, blank SLN and CUD-SLN are shown in Figure 2(C). The XRD patterns of blank SLN, and CUD-SLN were similar, and the multiple sharp peaks presented in the figure were consistent with the standard XRD pattern for sucrose (PDF 24-1977). CUD displayed three strong diffraction peaks at 13.640°, 15.921° and 20.581°, which disappeared in the diffraction patterns of CUD-SLN. The results showed that CUD was present as an amorphous form in CUD-SLN, which was consistent with the results of DSC. Taken together, these results confirmed that F68 and HSPC were good inclusion materials, thereby suggesting that SLN was the suitable carrier for CUD.

### *In vitro* drug release profile of CUD-SLN

**Figure 4.**
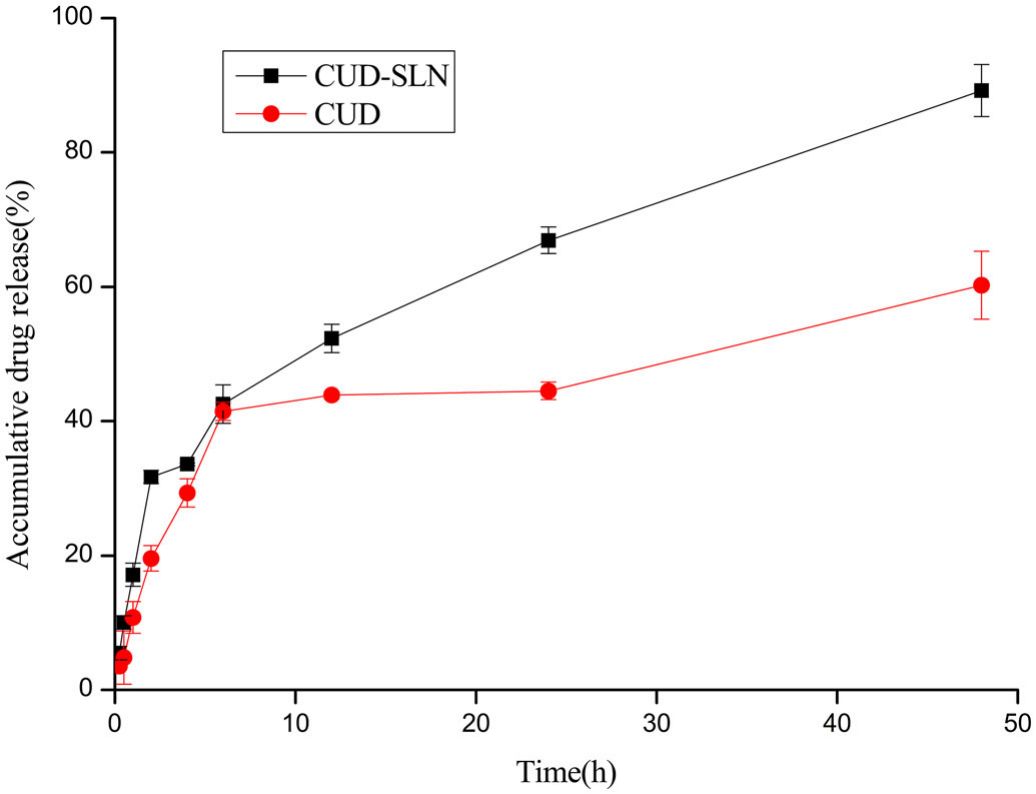
*In vitro* release curves of CUD suspension and CUD-SLN (*n* = 6).

To simulate the release of CUD-SLN and CUD suspensions, the release of CUD from SLN *in vitro* was studied by the dialysis membrane diffusion technique, and compared with CUD suspensions. The results are shown in Figure 4. The release rate of CUD-SLN was slightly faster than that released from the suspension, and at 48 h, CUD in the SLN was almost completely released (89.22 ± 3.39%), compared to the CUD in the suspension (60.24 ± 5.08%). To explore the release mechanism of the drug, the zero-order release equation, the first-order release equation and the Higuchi equation were employed to analyse the release data *in vitro*, and the kinetic parameters of the release rate are shown in Table 5. The release data of CUD were fit into the first-order release equation (*r*^2^=0.9458), while the release data of CUD-SLN were fit into Higuchi’s equation (*r*^2^=0.9624), as has been reported for drug-loaded SLN systems (Patlolla et al. 2010; Kakkar et al. 2018). From the curve fitting data, it was indicated that the release profile of CUD from CUD-SLN systems was diffusion-controlled.

**Table 5.** Dissolution kinetic parameters of CUD and CUD-SLN.

	Zero order equation		First-order equation		Higuchi’s equation	
	*r* ^2^	*k*_0_	*r* ^2^	*k*_1_	*r* ^2^	*k*_h_
CUD	0.6275	1.0410	0.9458	0.2258	0.8361	8.6574
CUD-SLN	0.8222	1.5861	0.8935	0.1335	0.9624	12.5234

The release rate of CUD was increased in CUD-SLN compared to free CUD. When F68 was exposed in the dissolution medium, the uniform dispersion of released drug particles in the medium was promoted as a non-ionic surfactant, and increase the solubility of the drug particles, thereby achieving improvement of the cumulative release of CUD-SLN (Arafat et al. 2021). Amorphous forms in the formulation can also ameliorate the release behaviour of the drug (Chono et al. 2008). According to the data obtained from XRD and DSC analysis, the existing form of CUD in CUD-SLN changes from crystalline to amorphous. Amorphous forms have large surface energy, which can improve the solubility of CUD-SLN. CUD-SLN showed satisfactory solubility in an aqueous environment (solubility >5 mg/mL at 25 °C), which laid the foundation for increasing the cumulative release.

### Pharmacokinetic studies

To study the pharmacokinetic behaviours of CUD-SLN, rats were injected with Cur and CUD-SLN at a dose of 5 mg/kg. As Cur is a water-insoluble drug, the preparation method of paclitaxel injection was used to prepare Cur injection as a control, namely, Cur was dissolved in a mixture of hydrogenated castor oil and ethanol (Rachmawati et al. 2018; Shi et al. 2019). CUD-SLN made from CUD was directly dissolved in normal saline before injecting because its solubility was significantly improved. Since CUD is difficult to dissolve in the above-mentioned solvents, free CUD cannot be effectively applied to injection methods. The purpose of this study was to prolong the duration of the drug action of Cur, and Cur was adopted as a control.

**Figure 5.**
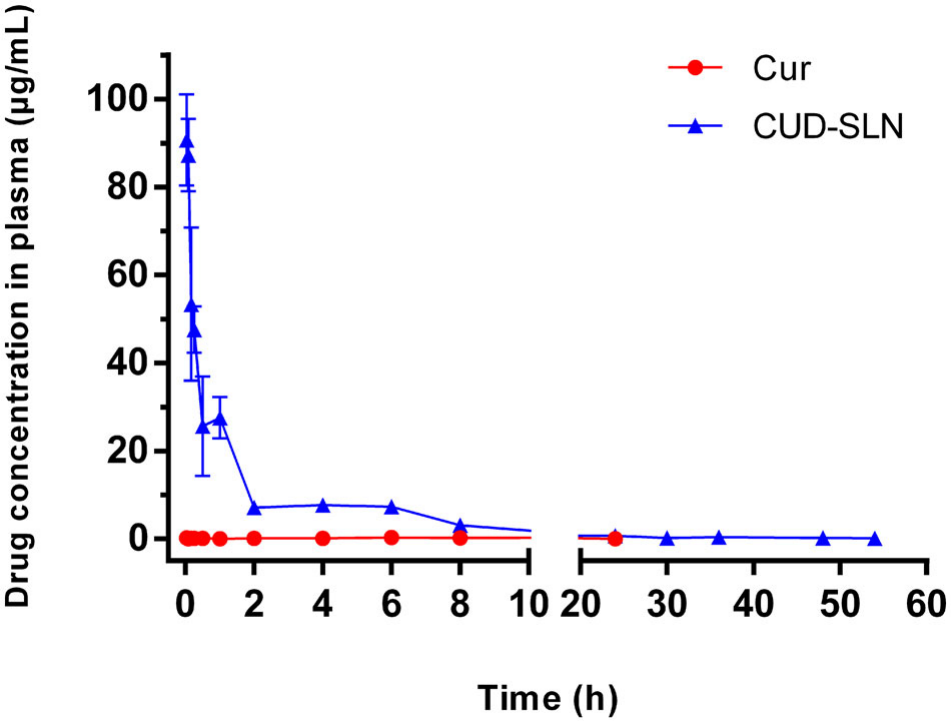
The plasma concentration–time curves of Cur and CUD-SLN (*n* = 5).

The plasma concentration–time curves are shown in Figure 5. The pharmacokinetic parameters are summarized in Table 6. When Cur was injected into the rat tail vein, the drug concentration did not peak immediately. The maximum concentration of Cur appeared later than expected, which could be related to the slow release of castor oil (Rachmawati et al. 2018). A significant difference was observed between Cur and CUD-SLN in a pharmacokinetic behaviour. The AUC_0–_*_t_* of CUD-SLN was 170.475 mg/L × h, which was 37.01-fold higher than that of Cur (4.605 mg/L × h), and was significantly increased. The clearance value of CUD-SLN in rats was 0.287 L/h/kg, which was nearly 35.67-fold lower than that of Cur (10.237 L/h/kg) (*p* < 0.05). In addition, the half-life (*t*_1/2_) of CUD-SLN was 14.774 h, which was about 2.64-fold that of Cur (5.6 h), and was significantly increased. Combined, these results indicated that the drug content in plasma was increased and that the existence of drugs in the systemic circulation was prolonged. This might be due to the physical and chemical properties of SLN, such as proper particle size and zeta potential, which reduced the uptake of macrophages by the mononuclear phagocytic system and increased delivery to target tissues (Li HJ et al. 2016; Kang et al. 2017). Cur-SLN with the use of HSPC as a membrane material has been reported. Compared with Cur, the AUC_0–_*_t_* only increased 9.02-fold after injection, while the AUC_0–_*_t_* of CUD-SLN increased 37.01-fold (Feng X et al. 2020). We speculate that the great stability of CUD also plays an important role in prolonging the duration of drug action.

**Table 6.** The pharmacokinetic parameters of Cur and CUD-SLN.

Parameters	Cur	CUD-SLN
AUC_0–_*_t_* (mg/L*h)	4.605	170.475
AUC_0–∞_ (mg/L*h)	4.884	174.245
MRT_0–_*_t_* (h)	9.529	7.736
MRT_0–∞_ (h)	10.817	9.328
*t*_1/2z_ (h)	5.600	14.774
*t*_max_ (h)	6.00	0.033
CLz (L/h/kg)	10.237	0.287
*C*_max_ (mg/L)	0.332	90.729

AUC_0–_*_t_*: area under the curve for 0 h to last time point; AUC_0–∞_: area under the whole curve; MRT_0–_*_t_*: mean retention time for 0 h to last time point; MRT_0–∞_: mean retention time for the whole curve; *t*_1/2z_: half-life time; *t*_max_: time to reach *C*_max_; CLz: clearance; *C*_max_: maximum drug concentration in plasma.

## Conclusions

The purpose of this study was to prolong the duration of drug action of Cur. In this study, CUD was used as the model drug, and F68 and HSPC were chosen as carriers to develop CUD-SLN. The results indicated that CUD-SLN has satisfactory solubility, release behaviour and pharmacokinetic behaviour. Therefore, CUD-SLN is a promising preparation for Cur in improving the stability *in vivo* and prolonging the duration of drug action. The study offers a methodology for the pharmaceutics research of Cur or flavonoid drugs. The efficacy of CUD-SLN warrants further investigation.

## References

[CIT0001] Alam M, Zameer S, Najmi AK, Ahmad FJ, Imam SS, Akhtar M. 2020. Thymoquinone loaded solid lipid nanoparticles demonstrated antidepressant-like activity in rats via indoleamine 2,3-dioxygenase pathway. Drug Res. 70(5):206–213. 10.1055/a-1131-7793 32198742

[CIT0002] Arafat M, Sarfraz M, AbuRuz S. 2021. Development and *in vitro* evaluation of controlled release Viagra(®) containing Poloxamer-188 using Gastroplus(™) PBPK modeling software for *in vivo* predictions and pharmacokinetic assessments. Pharmaceuticals. 14(5):479–489. 34070160 10.3390/ph14050479 PMC8158482

[CIT0003] Bagheri H, Ghasemi F, Barreto GE, Rafiee R, Sathyapalan T, Sahebkar A. 2020. Effects of curcumin on mitochondria in neurodegenerative diseases. Biofactors. 46(1):5–20. 31580521 10.1002/biof.1566

[CIT0004] Ban C, Jo M, Park YH, Kim JH, Han JY, Lee KW, Kweon DH, Choi YJ. 2020. Enhancing the oral bioavailability of curcumin using solid lipid nanoparticles. Food Chem. 302:125328. 31404868 10.1016/j.foodchem.2019.125328

[CIT0005] Cai X, Weng Q, Lin J, Chen G, Wang S. 2021. Radix Pseudostellariae protein–curcumin nanocomplex: improvement on the stability, cellular uptake and antioxidant activity of curcumin. Food Chem Toxicol. 151:112110. 33713747 10.1016/j.fct.2021.112110

[CIT0006] Chainoglou E, Hadjipavlou-Litina D. 2019. Curcumin analogues and derivatives with anti-proliferative and anti-inflammatory activity: structural characteristics and molecular targets. Expert Opin Drug Discov. 14(8):821–842. 31094233 10.1080/17460441.2019.1614560

[CIT0007] Chen S, Nimick M, Cridge AG, Hawkins BC, Rosengren RJ. 2018. Anticancer potential of novel curcumin analogs towards castrate-resistant prostate cancer. Int J Oncol. 52(2):579–588. 29207190 10.3892/ijo.2017.4207

[CIT0008] Chen XL, Liang XL, Zhao GW, Zeng QY, Dong W, Ou LQ, Zhang HN, Jiang QY, Liao ZG. 2021. Improvement of the bioavailability of curcumin by a supersaturatable self nanoemulsifying drug delivery system with incorporation of a hydrophilic polymer: *in vitro* and *in vivo* characterisation. J Pharm Pharmacol. 73(5):641–652. 33772289 10.1093/jpp/rgaa073

[CIT0009] Chono S, Takeda E, Seki T, Morimoto K. 2008. Enhancement of the dissolution rate and gastrointestinal absorption of pranlukast as a model poorly water-soluble drug by grinding with gelatin. Int J Pharm. 347(1–2):71–78. 17689212 10.1016/j.ijpharm.2007.06.037

[CIT0010] Dubey SK, Sharma AK, Narain U, Misra K, Pati U. 2008. Design, synthesis and characterization of some bioactive conjugates of curcumin with glycine, glutamic acid, valine and demethylenated piperic acid and study of their antimicrobial and antiproliferative properties. Eur J Med Chem. 43(9):1837–1846. 18201805 10.1016/j.ejmech.2007.11.027

[CIT0011] Feng T, Wei Y, Lee RJ, Zhao L. 2017. Liposomal curcumin and its application in cancer. Int J Nanomedicine. 12:6027–6044. 28860764 10.2147/IJN.S132434 PMC5573051

[CIT0012] Feng X, Pi C, Fu S, Yang H, Zheng X, Hou Y, Wang Y, Zhang X, Zhao L, Wei Y. 2020. Combination of curcumin and paclitaxel liposomes exhibits enhanced cytotoxicity towards A549/A549-T cells and unaltered pharmacokinetics. J Biomed Nanotechnol. 16(8):1304–1313. 33397559 10.1166/jbn.2020.2969

[CIT0013] Guo P, Pi C, Zhao S, Fu S, Yang H, Zheng X, Zhang X, Zhao L, Wei Y. 2020. Oral co-delivery nanoemulsion of 5-fluorouracil and curcumin for synergistic effects against liver cancer. Expert Opin Drug Deliv. 17(10):1473–1484. 32749895 10.1080/17425247.2020.1796629

[CIT0014] He Y, Zhan C, Pi C, Zuo Y, Yang S, Hu M, Bai Y, Zhao L, Wei Y. 2020. Enhanced oral bioavailability of felodipine from solid lipid nanoparticles prepared through effervescent dispersion technique. AAPS PharmSciTech. 21(5):170. 32529303 10.1208/s12249-020-01711-2

[CIT0016] Iqra R, Rizblabla M, Sadaf JG, May NB, Syed SI, Chandra K, Mohammad A, Sultan A, Satish KS. 2021. Thymoquinone loaded chitosan – solid lipid nanoparticles: formulation optimization to oral bioavailability study. J Drug Deliv Sci Technol. 64:102565.

[CIT0017] Kakkar V, Kaur IP, Kaur AP, Saini K, Singh KK. 2018. Topical delivery of tetrahydrocurcumin lipid nanoparticles effectively inhibits skin inflammation: *in vitro* and *in vivo* study. Drug Dev Ind Pharm. 44(10):1701–1712. 29938544 10.1080/03639045.2018.1492607

[CIT0018] Kang JH, Jang WY, Ko YT. 2017. The effect of surface charges on the cellular uptake of liposomes investigated by live cell imaging. Pharm Res. 34(4):704–717. 28078484 10.1007/s11095-017-2097-3

[CIT0019] Karade PG, Jadhav NR. 2018. Colon targeted curcumin microspheres laden with ascorbic acid for bioavailability enhancement. J Microencapsul. 35(4):372–380. 30010458 10.1080/02652048.2018.1501111

[CIT0020] Li HJ, Du JZ, Du XJ, Xu CF, Sun CY, Wang HX, Cao ZT, Yang XZ, Zhu YH, Nie S, et al. 2016. Stimuli-responsive clustered nanoparticles for improved tumor penetration and therapeutic efficacy. Proc Natl Acad Sci U S A. 113(15):4164–4169. 27035960 10.1073/pnas.1522080113 PMC4839420

[CIT0021] Li W, Li H, Yao H, Mu Q, Zhao G, Li Y, Hu H, Niu X. 2014. Pharmacokinetic and anti-inflammatory effects of sanguinarine solid lipid nanoparticles. Inflammation. 37(2):632–638. 24272172 10.1007/s10753-013-9779-8

[CIT0022] Lyu Y, Yu M, Liu Q, Zhang Q, Liu Z, Tian Y, Li D, Changdao M. 2020. Synthesis of silver nanoparticles using oxidized amylose and combination with curcumin for enhanced antibacterial activity. Carbohydr Polym. 230:115573. 31887939 10.1016/j.carbpol.2019.115573

[CIT0023] Ma Y, Wang Q, Wang D, Huang J, Sun R, Mao X, Tian Y, Xia Q. 2019. Silica-lipid hybrid microparticles as efficient vehicles for enhanced stability and bioaccessibility of curcumin. Food Technol Biotechnol. 57(3):319–330. 31866745 10.17113/ftb.57.03.19.6035 PMC6902299

[CIT0024] Maritim S, Boulas P, Lin Y. 2021. Comprehensive analysis of liposome formulation parameters and their influence on encapsulation, stability and drug release in glibenclamide liposomes. Int J Pharm. 592:120051. 33161039 10.1016/j.ijpharm.2020.120051

[CIT0025] Mishra S, Kapoor N, Mubarak Ali A, Pardhasaradhi BV, Kumari AL, Khar A, Misra K. 2005. Differential apoptotic and redox regulatory activities of curcumin and its derivatives. Free Radic Biol Med. 38(10):1353–1360. 15855053 10.1016/j.freeradbiomed.2005.01.022

[CIT0026] Mohammed SS, Rizblabla M, Syed SI. 2022. Formulation, optimization and evaluation of vitamin E TPGS emulsified dorzolamide solid lipid nanoparticles. J Drug Deliv Sci Technol. 68:103062.

[CIT0027] Momtazi-Borojeni AA, Haftcheshmeh SM, Esmaeili SA, Johnston TP, Abdollahi E, Sahebkar A. 2018. Curcumin: a natural modulator of immune cells in systemic lupus erythematosus. Autoimmun Rev. 17(2):125–135. 29180127 10.1016/j.autrev.2017.11.016

[CIT0028] Patlolla RR, Chougule M, Patel AR, Jackson T, Tata PN, Singh M. 2010. Formulation, characterization and pulmonary deposition of nebulized celecoxib encapsulated nanostructured lipid carriers. J Control Release. 144(2):233–241. 20153385 10.1016/j.jconrel.2010.02.006 PMC2868936

[CIT0029] Pawar H, Surapaneni SK, Tikoo K, Singh C, Burman R, Gill MS, Suresh S. 2016. Folic acid functionalized long-circulating co-encapsulated docetaxel and curcumin solid lipid nanoparticles: *in vitro* evaluation, pharmacokinetic and biodistribution in rats. Drug Deliv. 23(4):1453–1468. 26878325 10.3109/10717544.2016.1138339

[CIT0030] Rachmawati H, Arvin YA, Asyarie S, Anggadiredja K, Tjandrawinata RR, Storm G. 2018. Local sustained delivery of bupivacaine HCl from a new castor oil-based nanoemulsion system. Drug Deliv Transl Res. 8(3):515–524. 29516407 10.1007/s13346-018-0497-5

[CIT0031] Shi M, Gao T, Zhang T, Han H. 2019. Characterization of curcumin metabolites in rats by ultra-high-performance liquid chromatography with electrospray ionization quadrupole time-of-flight tandem mass spectrometry. Rapid Commun Mass Spectrom. 33(13):1114–1121. 30941839 10.1002/rcm.8450

[CIT0032] Slika L, Patra D. 2020. A short review on chemical properties, stability and nano-technological advances for curcumin delivery. Expert Opin Drug Deliv. 17(1):61–75. 31810374 10.1080/17425247.2020.1702644

[CIT0033] Tan MC, Matsuoka S, Ano H, Ishida H, Hirose M, Sato F, Sugiyama S, Murata M. 2014. Interaction kinetics of liposome-incorporated unsaturated fatty acids with fatty acid-binding protein 3 by surface plasmon resonance. Bioorg Med Chem. 22(6):1804–1808. 24581547 10.1016/j.bmc.2014.02.001

[CIT0034] Tian C, Asghar S, Hu Z, Qiu Y, Zhang J, Shao F, Xiao Y. 2019. Understanding the cellular uptake and biodistribution of a dual-targeting carrier based on redox-sensitive hyaluronic acid-ss-curcumin micelles for treating brain glioma. Int J Biol Macromol. 136:143–153. 31199976 10.1016/j.ijbiomac.2019.06.060

[CIT0038] Wei Y, Zeng M, Pi C, Shen H, Yuan J, Zuo Y, Wen J, Guo P, Zhao W, Li K, et al. 2022. Novel curcumin derivative-decorated ultralong-circulating paclitaxel nanoparticles: a novel delivery system with superior anticancer efficacy and safety. Int J Nanomedicine. 17:5265–5286. 36406640 10.2147/IJN.S369761 PMC9673813

[CIT0035] Wichitnithad W, Nimmannit U, Wacharasindhu S, Rojsitthisak P. 2011. Synthesis, characterization and biological evaluation of succinate prodrugs of curcuminoids for colon cancer treatment. Molecules. 16(2):1888–1900. 21343891 10.3390/molecules16021888 PMC6259653

[CIT0036] Yingchoncharoen P, Kalinowski DS, Richardson DR. 2016. Lipid-based drug delivery systems in cancer therapy: what is available and what is yet to come. Pharmacol Rev. 68(3):701–787. 27363439 10.1124/pr.115.012070 PMC4931871

[CIT0037] Zhao M, Zhao M, Fu C, Yu Y, Fu A. 2018. Targeted therapy of intracranial glioma model mice with curcumin nanoliposomes. Int J Nanomedicine. 13:1601–1610. 29588587 10.2147/IJN.S157019 PMC5858816

